# Protein-losing enteropathy caused by disseminated *Mycobacterium avium* complex infection in a patient receiving antiretroviral therapy: an autopsy case report

**DOI:** 10.1186/s12981-021-00417-0

**Published:** 2021-11-29

**Authors:** Keiji Konishi, Hidenori Nakagawa, Akio Nakahira, Takahiro Okuno, Takeshi Inoue, Michinori Shirano

**Affiliations:** 1 Department of Infectious Diseases, Osaka City General Hospital, 2-13-22 Miyakojimahondori, Miyakojima-ku, Osaka, Osaka 534-0021 Japan; 2 Department of Gastroenterology, Osaka City General Hospital, 2-13-22 Miyakojimahondori, Miyakojima-ku, Osaka, Osaka 534-0021 Japan; 3 Department of Diagnostic Pathology, Osaka City General Hospital, 2-13-22 Miyakojimahondori, Miyakojima-ku, Osaka, Osaka 534-0021 Japan

**Keywords:** Disseminated *Mycobacterium avium* complex infection, Protein-losing enteropathy, Antiretroviral therapy, Acquired immunodeficiency syndrome

## Abstract

**Background:**

Disseminated *Mycobacterium avium* complex infection is an important indicator of acquired immunodeficiency syndrome (AIDS) in patients with advanced human immunodeficiency virus (HIV) infection. Effective antiretroviral therapy has dramatically reduced the incidence of and mortality due to HIV infection, although drug resistance and poor medication adherence continue to increase the risk of disseminated *M. avium* complex infection. However, gastrointestinal lesions in cases of disseminated *M. avium* complex infection resulting in protein-losing enteropathy have been rarely discussed. Therefore, we present a case of protein-losing enteropathy caused by disseminated *M. avium* complex infection in a patient undergoing antiretroviral therapy.

**Case presentation:**

A 29-year-old man was diagnosed with AIDS 4 years ago and was admitted for a 10-month history of refractory diarrhea and fever. Despite receiving antiretroviral therapy, the viral load remained elevated due to poor medication adherence. The patient was diagnosed with disseminated *M. avium* complex infection and started on antimycobacterial drugs 2 years before admission. However, the infection remained uncontrolled. The previous hospitalization 1 year before admission was due to hypoalbuminemia and refractory diarrhea. Upper gastrointestinal endoscopy revealed a diagnosis of protein-losing enteropathy caused by intestinal lymphangiectasia, and treatment with intravenous antimycobacterial drugs did not resolve his intestinal lymphangiectasia. The patient inevitably died of sepsis.

**Conclusions:**

Clinical remission is difficult to achieve in patients with AIDS and protein-losing enteropathy caused by disseminated *M. avium* complex infection due to limited options of parenteral antiretroviral drugs. This report highlights the importance of identifying alternative treatments (such as an injectable formulation) for patients who do not respond to antiretroviral therapy due to protein-losing enteropathy with disseminated *M. avium* complex infection.

## Background

Among nontuberculous mycobacterial infections (e.g., those caused by *Mycobacterium avium* and *M. intracellulare*), disseminated *M. avium* complex (DMAC) infection is an important indicator of acquired immunodeficiency syndrome (AIDS) with an advanced human immunodeficiency virus (HIV) infection. The main symptoms of DMAC infection are fever and generalized lymph nodes, although gastrointestinal symptoms, such as diarrhea and abdominal pain, may also be present. Although there are only a few reports of protein-losing enteropathy (PLE) caused by DMAC infection, it is difficult to treat and has a poor prognosis. Herein, we present a case of PLE caused by DMAC infection in a patient with uncontrolled HIV infection due to poor drug absorption. Although it took 10 months from the onset of diarrhea to the diagnosis of PLE in the present case, the possibility of PLE caused by DMAC infection should be considered in patients with intractable diarrhea due to uncontrolled HIV infection.

## Case presentation

**Fig. 1:**
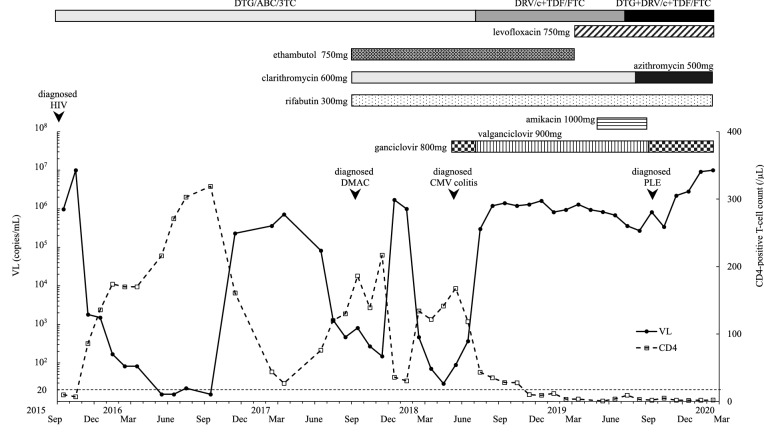
Timeline of drug and serial changes in CD4 cell count and HIV-1 RNA level. In the top table, the administered therapeutic drugs are mentioned. In the bottom table, the VL and CD4 cell count are indicated using open squares and closed circles, respectively. *HIV* human immunodeficiency virus, *DMAC* disseminated *Mycobacterium avium* complex, *CMV* cytomegalovirus, *PLE* protein-losing enteropathy, *VL* viral load, *DTG* dolutegravir, *ABC* abacavir, *3TC* lamivudine, *DRV* darunavir, *c* cobicistat, *TDF* tenofovir disoproxil fumarate, *FTC* emtricitabine

A 29-year-old man was admitted to our hospital in August 2019 for a 10-month history of refractory diarrhea and fever. In September 2015, he was diagnosed with HIV infection and AIDS following the onset of esophageal candidiasis, cytomegalovirus (CMV) enteritis, and *Pneumocystis jirovecii* pneumonia, which prompted the initiation of antiretroviral therapy (ART) with dolutegravir (DTG), abacavir, and lamivudine. However, viral replication persisted due to poor adherence to medications. Given that CMV enteritis is a frequent cause of medication malabsorption, the CMV antigen was measured and found to be suppressed. In May 2017, the patient was admitted to our hospital for fever associated with cervical and mediastinal lymphadenopathy with an elevated viral load (VL) of > 10 million copies/mL. Moreover, his CD4-positive T-lymphocyte count was low (17 cells/µL) (Fig. 1), and lymph node biopsy revealed the presence of *M. avium*. Findings were consistent with a diagnosis of DMAC infection, and despite receiving anti-mycobacterial therapy, such as clarithromycin, ethambutol, and rifabutin, his fever and lymphadenopathy did not improve. The antimicrobial susceptibility of the *M. avium* complex was also measured repeatedly, although the susceptibility of the *M. avium* complex to all drugs was maintained. This prompted the initiation of prednisolone for immune reconstitution inflammatory syndrome. In July 2018, his VL remained uncontrolled due to poor adherence to medications. Consequently, the ART was modified to tenofovir disoproxil fumarate (TDF), emtricitabine (FTC), darunavir (DRV), and cobicistat (c).

In October 2018, he presented with watery diarrhea; however, a colonoscopy revealed no abnormalities in the lower gastrointestinal tract. In March 2019, the patient also developed optic neuritis due to ethambutol. Treatment for DMAC infection was continued following the replacement of ethambutol with levofloxacin because antimicrobial blood cultures remained positive for *M. avium*. However, the patient’s fever and watery diarrhea worsened, and blood cultures remained positive for *M. avium* despite treatment. In May 2019, intravenous amikacin was initiated and later discontinued as the patient had symptoms of vomiting, sensorineural hearing loss, and progressive hypoalbuminemia. A colonoscopy showed an ulcer in the ileocecal region, and histopathologic analysis of the biopsy sample confirmed the diagnosis of CMV enteritis. Treatment with intravenous ganciclovir and oral maintenance therapy with valganciclovir did not help with his fever, watery diarrhea, and elevated VL. The HIV drug resistance test detected an M184V mutation, which led to further modification of the ART to TDF, FTC, DTG, and DRV/c in July 2019. However, VL remained elevated.

In August 2019, the patient was admitted to our hospital for monitoring of his refractory diarrhea, and a colonoscopy revealed improvement of the ulcers due to CMV enteritis. ^99m^Tc human serum albumin scintigraphy showed a faint accumulation in the center of the left upper quadrant at the third hour, followed by migration at the sixth hour. These findings were consistent with a diagnosis of PLE [1].

**Fig. 2:**
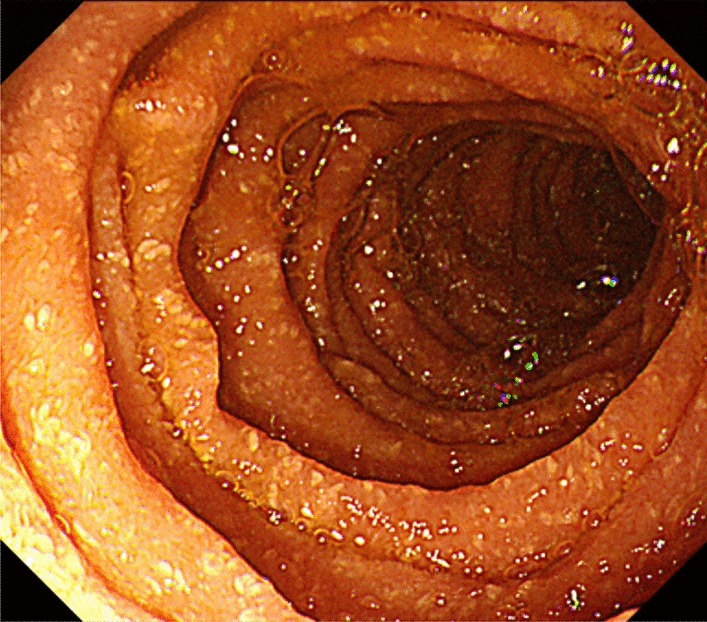
Findings of upper gastrointestinal endoscopy. Upper gastrointestinal endoscopy showing multiple yellowish-white granular nodules and lymphatic dilatation from the upper duodenal angle to the horizontal part of the duodenum

Capsule endoscopy revealed scattered yellowish-white nodules from the horizontal part of the duodenum to the upper jejunum, while upper gastrointestinal endoscopy (Fig. 2) showed multiple yellowish-white granular nodules and lymphatic dilatation from the upper duodenal angle to the horizontal part of the duodenum, suggesting that the PLE was due to intestinal lymphangiectasia (IL) [2]. Histopathologic analysis showed dense histiocytic infiltration in the mucosal lamina propria, while Ziehl–Neelsen staining showed numerous acid-fast bacteria. Polymerase chain reaction (PCR) identified the bacteria as *M. avium*. It was thought that the DMAC infection caused secondary IL, resulting in the development of PLE.

Since there was concern about poor antiretroviral drug (ARV) absorption in PLE, the plasma drug concentrations of DRV and DTG were measured. They were measured before (trough values) and after (peak values) oral administration; the plasma drug concentrations of both DRV and DTG were below the detection limit, indicating that the patient did not absorb any of his oral medications due to PLE. As a result, azithromycin, levofloxacin, ganciclovir, and prednisolone were administered intravenously, and ART (TDF, FTC, and DRV/c) was administered orally. Moreover, symptomatic therapy for splenomegaly and vomiting was also administered to the patient.

In February 2020, the patient was admitted to our hospital for worsening pain and hypotension, and as per the request of the patient and his family, cardiopulmonary resuscitation was not performed. He died due to sepsis on the third day after admission.

**Fig. 3:**
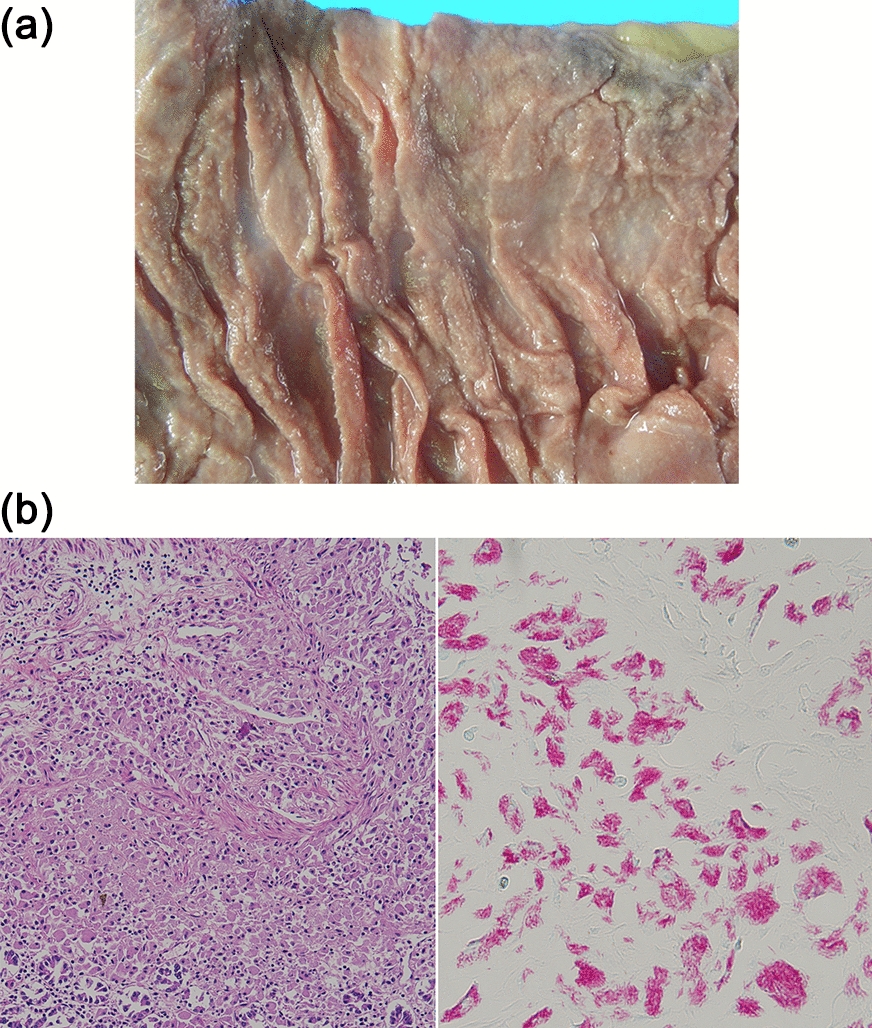
Gross pathologic findings and histopathologic examination. **a** Gross pathologic findings show white granular lesions that are diffusely present from the duodenum to the jejunum, and vasodilation is observed. **b** Histopathologic analysis show dense histiocytic infiltration in the mucosal lamina propria, and Ziehl–Neelsen staining showing numerous acid-fast bacilli (left, hematoxylin and eosin staining; right, Ziehl–Neelsen staining, both magnification ×200)

An autopsy was performed (Fig. 3) and revealed gross pathological findings as follows: (1) diffuse white granular esophageal and duodenojejunal lesions, (2) generalized intestinal edema, (3) enlarged and nodular peritoneal lymph nodes, and (4) an enlarged spleen (818 g) with similar white granular lesions. Histopathological examination showed acid-fast bacilli in various organs of the body, which includes the esophagus, stomach, duodenum, jejunum, ileum, colon, liver, spleen, lymph nodes (e.g., intestinal, periaortic, mediastinum), lung, adrenal gland, thyroid gland, bone marrow, and renal vessels. Particularly, the small intestine had diffuse lesions and was positive for the *M. avium* complex on PCR. Furthermore, a CMV infection was observed in the colon, lung, and adrenal glands, although the number of infected cells was small. Methicillin-susceptible *Staphylococcus aureus* was detected in the blood culture and masses in the right lung and bladder mucosa.

## Discussion and conclusions

We presented a case of PLE due to IL during ART in an adult Japanese man, and the etiology of PLE was known to be the obstruction of the intestinal and retroperitoneal lymphatic drainage due to *M. avium* complex lymphadenitis. As a result of PLE, the patient may have become unresponsive to oral medications, resulting in poor HIV control, which led to his death. However, the prognosis of DMAC infections has improved with the increasing use of ART, although the treatment of rare HIV cases with PLE due to DMAC infection remains challenging [3, 4].

DMAC infection is an important opportunistic infection in patients with advanced HIV, especially those with CD4-positive lymphocyte count < 50 cells/µL. From 1982 to 1994, the incidence of *M. avium* complex infections remained high before introducing highly active antiretroviral therapy (HAART) and prophylactic antibiotics. In the pre-HAART era, *M. avium* complex infection was a major cause of morbidity and mortality, affecting 15–24% of patients with AIDS [5], although after the introduction of HAART, the incidence of DMAC infections in HIV-infected patients decreased significantly. However, due to limited access to health care, resistance to ARVs, and poor medication adherence, the increased risk of *M. avium* complex infection continues [6].

The *M. avium* complex is transmitted via ingestion or inhalation, leading to colonization within the gastrointestinal or respiratory tract. Although colonization may or may not result in infection, these two processes often co-exist. The symptoms of DMAC infection are non-specific and may mimic other gastrointestinal-related illnesses. Therefore, isolation of the organism from tissues (e.g., blood, lymph nodes, liver, or bone marrow) is usually required to establish a diagnosis. Upper gastrointestinal endoscopy is an important diagnostic tool that should be considered in HIV-infected individuals with gastrointestinal symptoms if DMAC infection is suspected [7]. According to a report of endoscopic findings in DMAC infection, patients not treated with HAART were more likely to have endoscopic abnormalities than those treated with HAART [8]. Endoscopic findings include the following: (1) raised yellow or white plaques and nodules, (2) edema, (3) erosions and ulcers, (4) friability, and (5) decreased mucosal vascular pattern [8]. In the gastrointestinal tract, lesions in the duodenum (76%) were the most common, followed by those in the rectum (24%), ileum (6%), colon (4%), jejunum (2%), and stomach (2%) [8]. In this case, there were diffuse lesions in the small intestine consistent with PLE due to IL. To the best of our knowledge, *M. avium* complex infections with extensive gastrointestinal tract involvement while receiving ART are rarely reported. This study described a rare case of DMAC infection in an HIV-infected patient due to IL-induced PLE and poor medication adherence.

IL is a rare condition characterized by an obstruction of lymph vessels supplying the small intestines, potentially causing PLE, and classified into two types: primary and secondary. Primary IL usually occurs in children and adolescents due to an inherent lymphatic anomaly. In contrast, secondary IL occurs in adults due to an underlying disease (e.g., lymphoma, systemic lupus erythematosus, intestinal tuberculosis, retroperitoneal fibrosis, liver cirrhosis, constrictive pericarditis, and abdominal surgery). In our case, the patient had an IL secondary to *M. avium* complex infection lymphadenitis, obstructing the mesenteric and retroperitoneal lymphatic drainage. PLE may further aggravate the immunodeficiency due to the loss of T-lymphocytes and immunoglobulins (especially IgG) [9]. Therefore, the therapeutic management of PLE is necessary for patients with HIV infection. In the presence of secondary IL, treatment of the primary disease must be continued simultaneously. In our patient, antimycobacterial treatment was more aggressive. In addition to oral rifabutin and ethambutol, we also administered intravenous levofloxacin, azithromycin, and amikacin. However, despite this treatment, the infection persisted.

HIV-infected patients with malabsorption are difficult to manage. In some reports, the effective ART regimen for HIV-infected patients with malabsorption was determined using therapeutic drug monitoring (TDM) [10]. In our case, TDM of DTG and DRV was performed, which showed low levels of these drugs in the blood. Currently, all drugs used in ART listed in the guidelines are administered orally [11]. Effective ART drugs for HIV-infected patients with malabsorption are limited. In March 2020, the world’s first long-acting intramuscular formulation of cabotegravir and rilpivirine was approved in Canada, making the use of parenteral drugs possible. These drugs had sufficient blood levels with good antiretroviral efficacy when administered once every 1–2 months [12–14]. However, the intramuscular formulation in Canada is approved only for maintenance therapy in patients who have been successfully treated. Although parenteral drugs could have been the best option for the patient in our case, the intramuscular formulation is not approved for initial therapy.

We have described a rare case of DMAC due to IL-induced PLE and poor medication adherence in a Japanese patient with HIV infection. The emergence of drug-resistant viruses prompts further studies to determine the best therapeutic option for HIV-infected patients with PLE in terms of suitability, safety, and efficacy.

## Data Availability

The datasets used and analyzed during the current study are available from the corresponding author upon reasonable request.

## Abbreviations

DMAC: Disseminated *Mycobacterium avium* complex
AIDS: Autoimmune deficiency syndrome
HIV: Human immunodeficiency virus
PLE: Protein-losing enteropathy
CMV: Cytomegalovirus
ART: Antiretroviral therapy
DTG: Dolutegravir
VL: Viral load
TDF: Tenofovir disoproxil fumarate
FTC: Emtricitabine
DRV: Darunavir
c: Cobicistat
IL: Intestinal lymphangiectasia
PCR: Polymerase chain reaction
HAART: Highly active antiretroviral therapy
